# Understanding the complexities of space anaemia in extended space missions: revelations from microgravitational odyssey

**DOI:** 10.3389/fphys.2024.1321468

**Published:** 2024-03-11

**Authors:** Edouard Lansiaux, Nityanand Jain, Swarali Yatin Chodnekar, Abdelmomen Siddiq, Muiz Ibrahim, Mathieu Yèche, Inara Kantane

**Affiliations:** ^1^ Faculty of Medicine, Lille University School of Medicine, Lille, France; ^2^ Statistics Unit, Riga Stradinš University, Riga, Latvia; ^3^ Faculty of Medicine, Teaching University Geomedi LLC, Tbilisi, Georgia; ^4^ Faculty of Pharmacy, Mansoura University, Mansoura, Egypt; ^5^ Department of Public Health, International Higher School of Medicine, Bishkek, Kyrgyzstan; ^6^ Institut du Cerveau et de la Moelle épinière, Paris Brain Institute, Hopital de la Pitie-Salpetriere, Sorbonne Universite, INSERM U1127, CNRS UMR7225, Paris, France; ^7^ Département de Biologie de l’École Normale Supérieure (ENS), PSL Research University, Paris, France

**Keywords:** anaemia, spaceflight, space exploration, haemolysis, microgravity, radiation

## Abstract

Space travel exposes astronauts to several environmental challenges, including microgravity and radiation exposure. To overcome these stressors, the body undergoes various adaptations such as cardiovascular deconditioning, fluid shifts, metabolic changes, and alterations in the state of the bone marrow. Another area of concern is the potential impact of these adaptations on erythrocyte and haemoglobin concentrations, which can lead to what is commonly referred to as space anaemia or microgravity-induced anaemia. It is known that anaemia may result in impaired physical and cognitive performance, making early detection and management crucial for the health and wellbeing of astronauts during extended space missions. However, the effects and mechanisms of space anaemia are not fully understood, and research is underway to determine the extent to which it poses a challenge to astronauts. Further research is needed to clarify the long-term effects of microgravity on the circulatory system and to investigate possible solutions to address spaceflight-induced anaemia. This article reviews the potential link between spaceflight and anaemia, based on existing evidence from simulated studies (e.g., microgravity and radiation studies) and findings from spaceflight studies (e.g., International Space Station and space shuttle missions).

## 1 Introduction

Space exploration has significantly increased our understanding of the Solar System and beyond. However, space travel presents numerous physiological challenges for astronauts to overcome. A particularly significant challenge is space anaemia, also known as microgravity-induced anaemia or astronaut’s anaemia. As its name suggests, space anaemia is a type of anaemia that can develop due to prolonged exposure to microgravity during extended space missions. It is characterized by decreased levels of erythrocytes, haemoglobin, and/or haematocrit (Balakhovskiĭ and Noskov, 2008). Anaemia, regardless of its origin, can exert detrimental effects on oxygen transportation as well as cognitive and physical abilities (Agrawal et al., 2019).

Although, the exact mechanisms and consequences of this adaptation remain the subject of active study and debate, it is becoming increasingly recognized as a physiological adaptation to spaceflight (Smith, 2002). Given the growing frequency and duration of space missions, it is essential to conduct a comprehensive evaluation of the effects of space-induced anaemia.

Research is ongoing to understand space anaemia and to establish the groundwork for developing effective countermeasures that will ultimately safeguard the health of astronauts and promote mission success.

Hence, in this paper, we explore the existing evidence from simulated spaceflight studies and the findings published from astronauts who have been aboard the International Space Station (ISS) or on space shuttle missions.

## 2 RBC homeostasis

Red blood cells (RBCs) play an integral role in transporting oxygen and carbon dioxide throughout the body. They have a short lifespan of approximately 120 days and demonstrate limited ability to adapt to new environments and stressors (Phillips and Henderson, 2018). The erythrocyte lineage in bone marrow produces RBCs through a cascade stimulated by erythropoietin (Meck et al., 2001). RBCs exit the bone marrow 1 day before reaching full maturity. During this 24-h span, they are termed as reticulocytes and contain some organelles and ribosomal RNA. Clinically, the reticulocyte count can be used as an indicator of erythrocyte production (De Santo et al., 2005) and provides a direct measure of erythropoietic activity (Rai et al., 2023).

Considering the short lifespan of RBCs, our body aims to maintain a homeostatic equilibrium of circulating RBCs, by producing new precursor cells and destroying senescent erythrocytes at a constant rate, with imbalances leading to anaemia (Dinarelli et al., 2018). Mathematically, this can be expressed by equation *M = P x S*, where *M* represents the total mass of erythrocytes, *P* is the production rate of new erythrocytes, and *S* is the lifespan of erythrocytes (McKenzie et al., 2019).

## 3 Decline in RBC production

A reduction in RBC production arises from decreased erythroid precursor cells in the bone marrow, resulting in decreased RBC production (Keohane et al., 2020). Under normal gravity, there are several etiological factors that decrease RBC production, broadly classified into two groups–hereditary and acquired (Kaushansky et al., 2021). Alternatively, these factors can be grouped based on the pathophysiological mechanism - i) failure in the production of pluripotent hematopoietic stem cells; ii) failure in the differentiation of erythroid progenitor cells; and iii) dysfunction of progenitor cells due to nutrition-related problems.

It is now hypothesized that under conditions of spaceflight, a decrease in production of RBCs occurs as a compensatory mechanism rather than because of an underlying pathology (Tokarev and Andreeva, 1994).

This is evidenced by a decrease in the count of reticulocytes and other RBC indices including red blood cell mass (RBCM). This phenomenon has been observed not only in humans, but also in other mammals. A literature review conducted on published works prior to 1994, which included animal and human studies lasting between five and 12 months in space, compared samples taken before and after flight and reported a significant decrease in RBCM, reticulocyte count, haemoglobin, and erythropoietin (Tokarev and Andreeva, 1994). These findings could point towards a decreased RBC production resulting in anaemia.

Researchers and clinicians have attempted to explain this phenomenon through several potential mechanisms. The first hypothesis posited that the reduction of erythropoiesis in microgravity results from the decrease in sympathetic activity since autonomic mechanisms may regulate erythropoietin production (Robertson et al., 1994a; Robertson et al., 1994b; De Santo et al., 2005). The second hypothesis was based on a study in which rats were suspended to simulate microgravity conditions. Suspension in air resulted in suppressed erythropoiesis and a transient increase in haematocrit due to a reduction in plasma volume (Dunn et al., 1985). Another animal study with rats produced consistent results, and reported a decreased production of RBCs, as evidenced by low reticulocyte counts, and a decreased total RBC count in the marrow (Serova et al., 1993).

These findings have been consistently demonstrated in human studies as well. A study that measured blood indices during a spaceflight on the second day reported that erythropoietin levels were significantly lower, indicating a suppression in erythropoiesis (Leach et al., 1988). Another study showed a reduction in erythropoietin levels within 24 h of exposure to microgravity (Udden et al., 1995). It was noted that circulating RBCs were destroyed at a normal rate, but the rate of replacement was low, resulting in a decreased RBCM. The authors suggested that the cause could be *ineffective* erythropoiesis, rather than *insufficient* erythropoiesis, as the RBCs were not released at the rate of destruction (Udden et al., 1995). In a separate study that assessed RBC indices in short and long-term space travel, a reduced reticulocyte count and, consequently, a decline in RBCM were observed. The most likely explanation was a decrease in RBC production rather than an increase in haemolysis (Leach, 1992).

Low levels of erythropoietin were also observed due to microgravity-induced apoptosis and downregulation of erythropoietin receptors (Zoi et al., 2010). The decrease in erythropoietin levels correlated inversely with central venous pressure. This trend is also observable among individuals residing at high altitudes (Leach and Johnson, 1984; De Santo et al., 2005; Garrett-Bakelman et al., 2019). Epidemiological data indicated that the severity, time for recovery, and long-term effects of post-flight anaemia correspond to the duration of time spent in space (Kunz et al., 2017; Trudel et al., 2020).

## 4 Accelerated haemolysis of circulating RBCs

Nonetheless, the findings discussed in the previous section seem to counteract the results observed in spaceflight studies investigating the rate of haemolysis. The observed haemolysis patterns throughout the three phases of the study (pre-flight, in space, and post-flight) support a strong correlation between increased haemolysis and the conditions experienced in space (Rizzo et al., 2012). Premature red blood cell (RBC) destruction, known as haemolysis, can occur due to various reasons on Earth. Genetic defects, such as thalassemia or sickle cell disease, can cause haemolysis. RBCs can also be attacked by the complement system, resulting in intravascular destruction, as seen in autoimmune diseases or paroxysmal nocturnal haemoglobinuria. Haemolysis is also seen in HELLP syndrome during pre-eclampsia and in infections like malaria (Chaparro and Suchdev, 2019; Perez Botero et al., 2021). Haemolysis decreases the RBC count, reducing the oxygen and carbon dioxide transport capacity. As a result, a regenerative transient anaemia occurs, with an increased number of reticulocytes to compensate for the loss.

With a microgravity exposure of 10 days or longer, haematocrit levels begin to decrease. Normally, the human body creates and destroys two million erythrocytes per second under normal gravity. However, in microgravity, astronauts experience the destruction of three million erythrocytes per second. This expedited loss of RBCs was 54% higher than that of average people on Earth. On return to surface, RBCM, plasma volume, haematocrit, and reticulocyte number were reported to be consistently decreased (Leach and Johnson, 1984; De Santo et al., 2005; Garrett-Bakelman et al., 2019).

Concerning the possible risk factors inducing anaemia in space, the Gemini program proved that hyperoxia was at least partially responsible (Tavassoli, 1982). Despite this, anaemia has been seen to a lesser extent on subsequent space missions. Hence, haemolysis may be considered as the main cause of space anaemia.

A ground-based head-down tilt bed experiment simulating microgravity showed an increase in CO elimination and bilirubin levels in the blood (Culliton et al., 2021). Preferential haemolysis of recently produced RBCs has been observed during short missions, without alterations of pre-flight RBCs, a phenomenon known as “neocytolysis” (Alfrey et al., 1996; Alfrey et al., 1997). Furthermore, no discrepancies in RBC volume losses were noted between short and long duration spaceflight (Meck et al., 2001). To quantify the haemolysis, two biomarkers have been widely studied and reported in the studies–carbon monoxide (CO) elimination in exhaled alveolar air and levels of free haemoglobin in the blood (Landaw et al., 1970; Abbott, 2022).

Trudel et al., recently found a persistent increase in CO elimination over a 6-month period in space among 14 astronauts (Trudel et al., 2022). Post-flight measurements in the same study showed a rapid reversal of haemolysis markers 4 days after landing. This suggests that haemolysis’s consequences could not just be transient but might be more long-term. Haptoglobin, which binds to extracellular haemoglobin, promoting its safe clearance (Alayash et al., 2013), was also not decreased, indicating extravascular haemolysis. Additionally, mild haemolysis may not always result in anaemia, as demonstrated in a previous study where haemoglobin was only decreased by 11% (Minetti et al., 2022).

Nonetheless, the haemolytic phenomenon was also supported by elevated levels of haemolysis, reticulocytosis, and haemoglobin, suggesting that space-induced haemolytic anaemia is a primary effect of microgravity (Trudel et al., 2022). These values remained elevated during the first 7 days of exposure to microgravity, although there may be a slight decrease in reticulocyte counts. Space anaemia was characterized by a 10%–12% decrease in haematocrit within the first 10 days in space (Trudel et al., 2022). The occurrence of haemolytic anaemia was observed for the following year, and erythrocyte counts gradually returned to normal within three to 4 months. This indicates that extended space missions may lead to structural alterations that affect RBCs.

One year following landing, exhaled CO levels were thirty percent higher, haemoglobin concentration was three percent higher, and reticulocyte count was sixteen percent higher than pre-flight levels (Trudel et al., 2022). Accordingly, haemolysis that results in compensatory red blood cell synthesis raises the erythropoietic activity of the bone marrow. Excessive compensation may result in polycythaemia-haemolysis cycle. With individual microgravity exposures averaging 145 days, most of the data collected is limited to 1-year post-mission (Trudel et al., 2020). Based on these data, it has been modelled that it may take between 15 and 20 terrestrial years to fully overcome the negative effects of exposure to space (Trudel et al., 2020).

Apart from these two biomarkers, erythroid hyperplasia and reticulocytosis also serve as markers for all types of haemolytic anaemia. This is because reduced oxygen perfusion in tissues prompts increased erythropoietin production from the kidney, which in turn spurs erythroid element growth and release of reticulocytes from the marrow. Furthermore, in cases of severe anaemia, extramedullary haematopoiesis may occur in the liver, spleen, and lymph nodes (van Galen and Simsek, 2022). In microgravity, it has been proposed that a mixed form of aetiology may be responsible for haemolysis including altered size and shape of erythrocytes or the spleen (extravascular), mitochondrial stress and dysregulation (intra-corpuscular), and marrow adipose accumulation secondary to lack of bone stimulation, inadequate erythropoiesis, and changed methylation levels in CD4^+^ and CD8^+^ cells (extra-corpuscular) (Cogoli, 1981; Abbott, 2022).

Accelerated fat accumulation was observed in the bones responsible for producing blood during a microgravity simulation. Inactivity and lack of mechanical stimulation of bones can cause stem cells to promote bone or fat production in the marrow. In regular circumstances on Earth, the accumulation of fat in the marrow and changes in erythrocyte function and haemolysis are linked to aging. These intra-marrow changes appear irreversible under normal conditions. Furthermore, astronauts who spent more than a year in space exhibited a significant change in the methylation of CD4^+^ and CD8^+^. This implies that extended exposure to microgravity may lead to irreversible alterations in the blood clotting system (Garrett-Bakelman et al., 2019). However, the concrete correlations between reversibility/irreversibility of physiological changes in blood corpuscles and long-duration (>2 years) microgravity are yet to be studied (Rizzo et al., 2012). It remains unclear whether long-term exposure to microgravity can have an irreversible impact on bone marrow and erythrocyte production.

## 5 Structural changes in RBCs

RBC’s pivotal role of gas exchange is maintained through their distinct structure and shape, which allows for easy circulation within the blood vessels. The pliability of RBCs is crucial for their optimal performance, enabling them to manoeuvre through narrow capillary lumens (Ivanova et al., 2011). Microgravity has been shown to impact the structure and function of RBCs through various mechanisms, including induction of cytoskeletal and transcriptomic modifications. It is postulated that these changes may be introduced at the level of erythro-progenitors in the marrow in response to mechano-transduction forces and altered marrow chemistry (Ivanova et al., 2006).

Microgravity also causes cytosolic changes such as the breakdown of the total antioxidant capacity system, increased presence of reactive oxygen species (ROS), and a decrease in glutathione levels in RBCs (Bennett-Guerrero et al., 2007). At the same time, increased presence of phosphatidylcholine and nitric oxide may reduce RBC-dependent vasoregulation, as well as cause adenosine triphosphate (ATP) response abolition and an increase in RBC deformability (Bennett-Guerrero et al., 2007). In a study investigating blood samples from astronauts during space flight, an increase in phosphatidylcholine was reported, leading to increased rigidity of the RBC membrane system (Ivanova et al., 2006). This rigidity could be harmful to the function of RBCs, which require a certain level of fluidity to perform their necessary functions within the human body (Boonstra, 1999).

Under microgravity conditions, sphingomyelin has been shown to increase by a 42:1 ratio with corresponding increase in the ROS levels. This correlation of sphingomyelin and ROS increase was associated with an acquired inflammatory state (Manis et al., 2022), inducing changes into RBC cytoskeletal architecture and membrane characteristics (stiffness, microviscosity and permeability).

The membrane of erythrocytes may undergo morphological changes resulting from decreased fluidity caused by elevated cholesterol levels. It was shown that the decrease in membrane fluidity was accompanied by an upsurge in Na^+^/H^+^ exchanger and K^+^/Ca2^+^ channels activity (Manis et al., 2022). The rise in Na^+^/H^+^ exchanger activity appeared following elevation in cholesterol concentration in erythrocyte membranes due to microgravity.

This could result in modified lipid distribution and changes in membrane and ion channel permeabilities, as well as oxidative stress (Sheetz and Singer, 1974; Wong, 1999). The presence of RBCs in an oxidative stress state has been linked to lipid peroxidation, leading to elevated diene conjugates while superoxide dismutase and tocopherol levels decrease, ultimately resulting in a changed membrane morphology (Ivanova et al., 2011). Furthermore, the reduction in fluidity of the RBCs could negatively impact the oxygen and carbon dioxide exchange function due to the decrease of total haemoglobin and oxyhaemoglobin. However, this can be offset by an increase in the haemoglobin’s oxygen affinity. Nonetheless, it has been reported that these effects may be reversible after 2 weeks under normal gravity (Ivanova et al., 2011).

## 6 Fluid shift and decreased plasma volume

As astronauts enter the microgravity environment, hydrostatic pressure and levels of physical activity decrease. This causes a shift of fluid to the upper body, resulting in a transient increase in thoraco-pulmonary vascular and cardiac stroke volumes. The increased volume distends the central vasculature, stimulating the central carotid, aortic, and cardiac receptors to decrease fluid overload (Iwase et al., 2020).

Furthermore, fluid shift leads to an initial 20% increase in the size of heart chambers which, in turn, results in an 80% increase in atrial natriuretic peptide (ANP) secretion levels within the first day of microgravity, causing vasodilation and extravasation of fluid and sodium (Aubert et al., 2005; Bureau et al., 2017).

In addition, reduced fluid intake due to decreased angiotensin levels and loss of muscle tone in microgravity are thought to contribute to this extravasation (Grigoriev et al., 1994). This leads to characteristic symptoms such as a swollen face, blocked nose, and chicken legs (Ertl et al., 2002; Aubert et al., 2005). Consequently, short-term exposure to microgravity, i.e., less than 10 days, results in a 10%–15% reduction in plasma volume and atrial pressure (Aubert et al., 2005; Pavy-Le Traon et al., 2007). However, prolonged microgravity exposure has been shown to be associated with a 10% decrease in ventricular size within the initial 24–48 h following the spaceflight. These changes are in addition to microgravity-induced decreases in metabolic demand and oxygen uptake, which can lead to cardiac atrophy by 8%–10%. The heart’s structure becomes more spherical post-flight, but from day three onwards, size and structure changes may return to normal (Blomqvist, 1996; Summers et al., 2005). Several reports from space flights have reported on the incidence of arrhythmias, including atrial and ventricular premature contractions, short-duration atrial fibrillation, and non-sustained ventricular tachycardia among astronauts (Baran et al., 2021).

The analysis from 19 astronauts on a space shuttle mission showed that the increase in heart volumes was proportional to the amount of fluid shift (Simanonok and Charles, 1994). This fluid shift, along with plasma volume loss-mediated haemoconcentration and ANP release, induced neuro-vestibular disturbances that led to space sickness, nausea, and vomiting (Kaushansky et al., 2021). It was observed that post-flight the decrease in astronaut’s heart volume directly correlated with the severity of the sickness symptoms (Simanonok and Charles, 1994). Finally, microgravity-induced losses in plasma volume and altered autonomic functioning have been shown to cause orthostatic intolerance (OI) in astronauts, especially after short-duration spaceflights (Buckey et al., 1996; Fritsch-Yelle et al., 1996). A pilot investigation exploring the prevalence of OI after longer spaceflights ranging from four to 6 months furthered these findings by reporting a nearly five-fold increase in OI incidence when comparing long-term with short-term spaceflight (Meck et al., 2001).

Therefore, exposure to microgravity leads to a decrease in plasma volume, altered cardiac function, weight loss, and space sickness. Furthermore, microgravity can result in reduced production of erythropoietin and other growth factors (De Santo et al., 2005). It can be suggested that the cascading effect of shift in volume and loss in plasma volume leads to a decrease in RBC production due to haemoconcentration. In fact, it was found that upon return to Earth, anaemia and hypovolemia became prominent as compensatory mechanisms reversed (Alfrey et al., 1996). An *in vitro* study examined the proliferation of hematopoietic progenitor cells and found a decrease in the proliferation of these cells in microgravity when compared to normal ground gravity controls, suggesting a possible mechanism for the reduced production of RBCs (Davis et al., 1996).

## 7 Conclusion

Space anaemia, which is characterized by a reduction in the number of red blood cells and the concentration of haemoglobin, is a complex and multi-factorial condition that affects astronauts on long-duration space missions. The condition’s development is influenced by several factors, including fluid shift, decreased red blood cell production, and RBC sequestration. Effective interventions for space anaemia are presently limited to exercise and pharmaceuticals. The evidence suggests a requirement for additional research to determine effective and safe countermeasures for space anaemia. Furthermore, close collaboration will be required among space agencies, medical, and scientific communities to ensure that the latest research advancements are translated into practical solutions for astronauts.

## Data Availability

The original contributions presented in the study are included in the article/Supplementary material, further inquiries can be directed to the corresponding author.

## References

[B1] Abbott B. (2022). A mission to understand space anaemia. Commun. Med. (Lond). 2, 16. 10.1038/s43856-022-00078-8 35603286 PMC9053183

[B2] Agrawal S. Kumar S. Ingole V. Acharya S. Wanjari A. Bawankule S. (2019). Does anemia affects cognitive functions in neurologically intact adult patients: two year cross sectional study at rural tertiary care hospital. J. Fam. Med. Prim. Care 8 (9), 3005–3008. 10.4103/jfmpc.jfmpc_599_19 PMC6820398 31681682

[B3] Alayash A. I. Andersen C. B. Moestrup S. K. Bülow L. (2013). Haptoglobin: the hemoglobin detoxifier in plasma. Trends Biotechnol. 31 (1), 2–3. 10.1016/j.tibtech.2012.10.003 23140673

[B4] Alfrey C. P. Rice L. Udden M. M. Driscoll T. B. (1997). Neocytolysis: physiological down-regulator of red-cell mass. Lancet 349 (9062), 1389–1390. 10.1016/S0140-6736(96)09208-2 9149714

[B5] Alfrey C. P. Udden M. M. Leach-Huntoon C. Driscoll T. Pickett M. H. (1996). Control of red blood cell mass in spaceflight. J. Appl. Physiol. 81 (1), 98–104. 10.1152/jappl.1996.81.1.98 8828651

[B6] Aubert A. E. Beckers F. Verheyden B. (2005). Cardiovascular function and basics of physiology in microgravity. Acta Cardiol. 60 (2), 129–151. 10.2143/AC.60.2.2005024 15887469

[B7] Balakhovskiĭ I. S. Noskov V. B. (2008). Hemoglobin mass in humans during space flight and its simulation. Aviakosm Ekol. Med. 42 (6), 32–44. 19238915

[B8] Baran R. Marchal S. Garcia Campos S. Rehnberg E. Tabury K. Baselet B. (2021). The cardiovascular system in space: focus on *in vivo* and *in vitro* studies. Biomedicines 10 (1), 59. 10.3390/biomedicines10010059 35052739 PMC8773383

[B9] Bennett-Guerrero E. Veldman T. H. Doctor A. Telen M. J. Ortel T. L. Reid T. S. (2007). Evolution of adverse changes in stored RBCs. Proc. Natl. Acad. Sci. U. S. A. 104 (43), 17063–17068. 10.1073/pnas.0708160104 17940021 PMC2040393

[B10] Blomqvist G. C. (1996). Regulation of the systemic circulation at microgravity and during readaptation to 1G. Med. Sci. Sports Exerc 28 (10 Suppl. l), S9–S13. 10.1097/00005768-199610000-00025 8897396

[B11] Boonstra J. (1999). Growth factor-induced signal transduction in adherent mammalian cells is sensitive to gravity. FASEB J. 13 (Suppl. l), S35–S42. 10.1096/fasebj.13.9001.s35 10352143

[B12] Buckey J. C. Jr Lane L. D. Levine B. D. Watenpaugh D. E. Wright S. J. Moore W. E. (1996). Orthostatic intolerance after spaceflight. J. Appl. Physiol. 81 (1), 7–18. 10.1152/jappl.1996.81.1.7 8828642

[B13] Bureau L. Coupier G. Dubois F. Duperray A. Farutin A. Minetti C. (2017). Blood flow and microgravity. Comptes Rendus Mec. 345 (1), 78–85. 10.1016/j.crme.2016.10.011

[B14] Chaparro C. M. Suchdev P. S. (2019). Anemia epidemiology, pathophysiology, and etiology in low- and middle-income countries. Ann. N. Y. Acad. Sci. 1450 (1), 15–31. 10.1111/nyas.14092 31008520 PMC6697587

[B15] Cogoli A. (1981). Hematological and immunological changes during space flight. Acta astronaut. 8 (9-10), 995–1002. 10.1016/0094-5765(81)90070-9 11543118

[B16] Culliton K. Louati H. Laneuville O. Ramsay T. Trudel G. (2021). Six degrees head-down tilt bed rest caused low-grade hemolysis: a prospective randomized clinical trial. NPJ Microgravity 7 (1), 4. 10.1038/s41526-021-00132-0 33589644 PMC7884785

[B17] Davis T. A. Wiesmann W. Kidwell W. Cannon T. Kerns L. Serke C. (1996). Effect of spaceflight on human stem cell hematopoiesis: suppression of erythropoiesis and myelopoiesis. J. Leukoc. Biol. 60 (1), 69–76. 10.1002/jlb.60.1.69 8699125

[B18] De Santo N. G. Cirillo M. Kirsch K. A. Correale G. Drummer C. Frassl W. (2005). “Anemia and erythropoietin in space flights,” in Seminars in nephrology (WB Saunders), 25, 379–387. 16298259 10.1016/j.semnephrol.2005.05.006

[B19] Dinarelli S. Longo G. Dietler G. Francioso A. Mosca L. Pannitteri G. (2018). Erythrocyte's aging in microgravity highlights how environmental stimuli shape metabolism and morphology. Sci. Rep. 8 (1), 5277. 10.1038/s41598-018-22870-0 29588453 PMC5869709

[B20] Dunn C. D. Johnson P. C. Lange R. D. Perez L. Nessel R. (1985). Regulation of hematopoiesis in rats exposed to antiorthostatic, hypokinetic/hypodynamia: I. Model description. Aviat. Space Environ. Med. 56 (5), 419–426. 4004676

[B21] Ertl A. C. Diedrich A. Biaggioni I. Levine B. D. Robertson R. M. Cox J. F. (2002). Human muscle sympathetic nerve activity and plasma noradrenaline kinetics in space. J. Physiol. 538 (Pt 1), 321–329. 10.1113/jphysiol.2001.012576 11773339 PMC2290013

[B22] Fritsch-Yelle J. M. Whitson P. A. Bondar R. L. Brown T. E. (1996). Subnormal norepinephrine release relates to presyncope in astronauts after spaceflight. J. Appl. Physiol. (1985) 81 (5), 2134–2141. 10.1152/jappl.1996.81.5.2134 8941538

[B23] Garrett-Bakelman F. E. Darshi M. Green S. J. Gur R. C. Lin L. Macias B. R. (2019). The NASA Twins Study: a multidimensional analysis of a year-long human spaceflight. Science 364 (6436), eaau8650. eaau8650. 10.1126/science.aau8650 30975860 PMC7580864

[B24] Grigoriev A. I. Morukov B. V. Vorobiev D. V. (1994). Water and electrolyte studies during long-term missions onboard the space stations SALYUT and MIR. Clin. Investig. 72 (3), 169–189. 10.1007/BF00189308 8012159

[B25] Ivanova S. M. Brazhe N. A. Luneva O. G. Yarlikova Y. V. Labetskaya O. I. Parshina E. Y. (2011). Physical–chemical properties of plasma membrane and function of erythrocytes of cosmonauts after long-term space flight. Acta Astronaut. 68 (9-10), 1517–1522. 10.1016/j.actaastro.2010.06.046

[B26] Ivanova S. M. Morukov B. V. Labetskaia O. I. Iarlykova I. V. Levina A. A. Kozinets G. I. (2006). Morphobiochemical assay of the red blood system in members of the prime crews of the International Space Station. Aviakosm Ekol. Med. 40 (3), 9–15. 17193962

[B27] Iwase S. Nishimura N. Tanaka K. Mano T. (2020). “Effects of microgravity on human physiology,” in Beyond LEO - human health issues for deep space exploration. Editor Reynolds R. J. (London, UK: Intech Open), 1–22. 10.5772/intechopen.90700

[B28] Kaushansky K. Lichtman M. Prchal J. Levi M. Burns L. Linch D. C. (2021). Williams hematology. 10th Edition. New York City, NY, United States: McGraw Hill, 1–2704.

[B29] Keohane E. M. Otto C. N. Walenga J. M. (2020). Rodak's hematology - clinical principles and applications. 6th Edition. Amsterdam, Netherlands: Saunders Elsevier, 62–77.

[B30] Kunz H. Quiriarte H. Simpson R. J. Ploutz-Snyder R. McMonigal K. Sams C. (2017). Alterations in hematologic indices during long-duration spaceflight. BMC Hematol. 17, 12. 10.1186/s12878-017-0083-y 28904800 PMC5590186

[B31] Landaw S. A. Callahan E. W. Jr Schmid R. (1970). Catabolism of heme *in vivo*: comparison of the simultaneous production of bilirubin and carbon monoxide. J. Clin. Invest. 49 (5), 914–925. 10.1172/JCI106311 5441545 PMC535764

[B32] Leach C. S. (1992). Biochemical and hematologic changes after short-term space flight. Microgravity Q. 2 (2), 69–75. 11537105

[B33] Leach C. S. Johnson P. C. (1984). Influence of spaceflight on erythrokinetics in man. Science 225 (4658), 216–218. 10.1126/science.6729477 6729477

[B34] Leach C. S. Johnson P. C. Cintrón N. M. (1988). The endocrine system in space flight. Acta Astronaut. 17 (2), 161–166. 10.1016/0094-5765(88)90017-3 11537094

[B35] Manis C. Manca A. Murgia A. Uras G. Caboni P. Congiu T. (2022). Understanding the behaviour of human cell types under simulated microgravity conditions: the case of erythrocytes. Int. J. Mol. Sci. 23 (12), 6876. 10.3390/ijms23126876 35743319 PMC9224527

[B36] McKenzie S. B. Landis-Piwowar K. Bergeron J. D. Williams L. (2019). Clinical laboratory hematology. 4th edition. London, United Kingdom: Pearson, 1–1081.

[B37] Meck J. V. Reyes C. J. Perez S. A. Goldberger A. L. Ziegler M. G. (2001). Marked exacerbation of orthostatic intolerance after long-vs short-duration spaceflight in veteran astronauts. Psychosom. Med. 63 (6), 865–873. 10.1097/00006842-200111000-00003 11719623

[B38] Minetti G. Bogdanova A. Y. Mairbäurl H. Kaestner L. (2022). Space anemia unexplained: red blood cells seem to be space-proof. Am. J. Hematol. 97 (10), E365–E367. 10.1002/ajh.26663 35836385

[B39] Pavy-Le Traon A. Heer M. Narici M. V. Rittweger J. Vernikos J. (2007). From space to Earth: advances in human physiology from 20 years of bed rest studies (1986-2006). Eur. J. Appl. Physiol. 101 (2), 143–194. 10.1007/s00421-007-0474-z 17661073

[B40] Perez Botero J. Reese J. A. George J. N. McIntosh J. J. (2021). Severe thrombocytopenia and microangiopathic hemolytic anemia in pregnancy: a guide for the consulting hematologist. Am. J. Hematol. 96 (12), 1655–1665. 10.1002/ajh.26328 34424560 PMC8616841

[B41] Phillips J. Henderson A. C. (2018). Hemolytic anemia: evaluation and differential diagnosis. Am. Fam. Physician 98 (6), 354–361. 30215915

[B42] Rai D. Wilson A. M. Moosavi L. (2023). Histology, reticulocytes. Treasure Island (FL): StatPearls Publishing. [Updated 2023 May 19]. In: StatPearls https://www.ncbi.nlm.nih.gov/books/NBK542172/. 31194329

[B43] Rizzo A. M. Corsetto P. A. Montorfano G. Milani S. Zava S. Tavella S. (2012). Effects of long-term space flight on erythrocytes and oxidative stress of rodents. PLoS one 7 (3), e32361. 10.1371/journal.pone.0032361 22412864 PMC3296700

[B44] Robertson D. Convertino V. A. Vernikos J. (1994b). The sympathetic nervous system and the physiologic consequences of spaceflight: a hypothesis. Am. J. Med. Sci. 308 (2), 126–132. 10.1097/00000441-199408000-00014 8042655

[B45] Robertson D. Krantz S. B. Biaggioni I. (1994a). The anemia of microgravity and recumbency: role of sympathetic neural control of erythropoietin production. Acta Astronaut. 33, 137–141. 10.1016/0094-5765(94)90118-x 11539513

[B46] Serova L. V. Chelńaia N. A. Ivanova S. I. (1993). Comparative analysis of weightlessness and hypergravity effects on erythropoiesis in male and female mammals. Aviakosm Ekol. Med. 27 (1), 54–59. 8220342

[B47] Sheetz M. P. Singer S. J. (1974). Biological membranes as bilayer couples. A molecular mechanism of drug-erythrocyte interactions. Proc. Natl. Acad. Sci. U. S. A. 71 (11), 4457–4461. 10.1073/pnas.71.11.4457 4530994 PMC433905

[B48] Simanonok K. E. Charles J. B. (1994). Space sickness and fluid shifts: a hypothesis. J. Clin. Pharmacol. 34 (6), 652–663. 10.1002/j.1552-4604.1994.tb02020.x 8083397

[B49] Smith S. M. (2002). Red blood cell and iron metabolism during space flight. Nutrition 18 (10), 864–866. 10.1016/s0899-9007(02)00912-7 12361780

[B50] Summers R. L. Martin D. S. Meck J. V. Coleman T. G. (2005). Mechanism of spaceflight-induced changes in left ventricular mass. Am. J. Cardiol. 95 (9), 1128–1130. 10.1016/j.amjcard.2005.01.033 15842991

[B51] Tavassoli M. (1982). Anemia of spaceflight. Blood 60 (5), 1059–1067. PMID: 7126864. 10.1182/blood.v60.5.1059.1059 7126864

[B52] Tokarev I. N. Andreeva A. P. (1994). Pathogenetic mechanisms of the so-called astronauts' anemia. Gematol. Transfuziol. 39 (4), 17–21. 7875514

[B53] Trudel G. Shafer J. Laneuville O. Ramsay T. (2020). Characterizing the effect of exposure to microgravity on anemia: more space is worse. Am. J. Hematol. 95 (3), 267–273. 10.1002/ajh.25699 31816115

[B54] Trudel G. Shahin N. Ramsay T. Laneuville O. Louati H. (2022). Hemolysis contributes to anemia during long-duration space flight. Nat. Med. 28 (1), 59–62. 10.1038/s41591-021-01637-7 35031790 PMC8799460

[B55] Udden M. M. Driscoll T. B. Pickett M. H. Leach-Huntoon C. S. Alfrey C. P. (1995). Decreased production of red blood cells in human subjects exposed to microgravity. J. Lab. Clin. Med. 125 (4), 442–449. 7706898

[B56] van Galen L. S. Simsek S. (2022). Chilling to the marrow: finding extramedullary haematopoiesis in an unusual location behind the xiphoid. Lancet 400 (10357), e9. 10.1016/S0140-6736(22)01287-9 36154690

[B57] Wong P. (1999). A basis of echinocytosis and stomatocytosis in the disc-sphere transformations of the erythrocyte. J. Theor. Biol. 196 (3), 343–361. 10.1006/jtbi.1998.0845 10049626

[B58] Zoi L.-X. Cui S.-Y. Zhong J. Yi Z.-C. Sun Y. Fan Y.-B. (2010). Simulated microgravity induce apoptosis and down-regulation of erythropoietin receptor of UT-7/EPO cells. Adv. Space Res. 46 (10), 1237–1244. 10.1016/j.asr.2010.06.037

